# Rational metabolic engineering of Corynebacterium glutamicum to create a producer of L-valine

**DOI:** 10.18699/VJGB-22-90

**Published:** 2022-12

**Authors:** M.E. Sheremetieva, K.E. Anufriev, T.M. Khlebodarova, N.A. Kolchanov, A.S. Yanenko

**Affiliations:** NRC “Kurchatov Institute”, Kurchatov Genomic Center, Moscow, Russia; NRC “Kurchatov Institute”, Kurchatov Genomic Center, Moscow, Russia; Institute of Cytology and Genetics of the Siberian Branch of the Russian Academy of Sciences, Novosibirsk, Russia Kurchatov Genomic Center of ICG SB RAS, Novosibirsk, Russia; Institute of Cytology and Genetics of the Siberian Branch of the Russian Academy of Sciences, Novosibirsk, Russia Kurchatov Genomic Center of ICG SB RAS, Novosibirsk, Russia; NRC “Kurchatov Institute”, Kurchatov Genomic Center, Moscow, Russia

**Keywords:** Corynebacterium glutamicum, L-valine, metabolic engineering, producer strain, Corynebacterium glutamicum, L-валин, метаболическая инженерия, штамм-продуцент

## Abstract

L-Valine is one of the nine amino acids that cannot be synthesized de novo by higher organisms and must come from food. This amino acid not only serves as a building block for proteins, but also regulates protein and energy metabolism and participates in neurotransmission. L-Valine is used in the food and pharmaceutical industries, medicine and cosmetics, but primarily as an animal feed additive. Adding L-valine to feed, alone or mixed with other essential amino acids, allows for feeds with lower crude protein content, increases the quality and quantity of pig meat and broiler chicken meat, as well as improves reproductive functions of farm animals. Despite the fact that the market for L-valine is constantly growing, this amino acid is not yet produced in our country. In modern conditions, the creation of strains-producers and organization of L-valine production are especially relevant for Russia. One of the basic microorganisms most commonly used for the creation of amino acid producers, along with Escherichia coli, is the soil bacterium Corynebacterium glutamicum. This review is devoted to the analysis of the main strategies for the development of L- valine producers based on C. glutamicum. Various aspects of L-valine biosynthesis in C. glutamicum are reviewed: process biochemistry, stoichiometry and regulation, enzymes and their corresponding genes, export and import systems, and the relationship of L-valine biosynthesis with central cell metabolism. Key genetic elements for the creation of C. glutamicum-based strains-producers are identified. The use of metabolic engineering to enhance L-valine biosynthesis reactions and to reduce the formation of byproducts is described. The prospects for improving strains in terms of their productivity and technological characteristics are shown. The information presented in the review can be used in the production of producers of other amino acids with a branched side chain, namely L-leucine and L-isoleucine, as well as D-pantothenate.

## Abstract (ru)

L-Валин – одна из девяти аминокислот, которые не могут быть синтезированы de novo высшими организмами и должны поступать с пищей. Эта аминокислота не только служит строительным материалом для белков, но также регулирует белковый и энергетический обмен и участвует в нейротрансмиссии. L-Валин используется в пищевой и фармацевтической промышленности, медицине и косметике, но в первую очередь в качестве кормовой добавки для животных. Добавление L-валина в корм отдельно или в смеси с другими незаменимыми аминокислотами позволяет использовать корма с меньшим содержанием сырого белка, повышает качество и количество мяса свиней и цыплят-бройлеров, а также улучшает репродуктивные функции сельскохозяйственных животных. Несмотря на то что рынок L-валина постоянно растет, в нашей стране эта аминокислота пока не производится. В современных условиях создание штаммов-продуцентов и организация производства L-валина для России особенно актуальны. Один из наиболее часто используемых базовых микроорганизмов для создания продуцентов аминокислот наряду с Escherichia coli – почвенная бактерия Corynebacterium glutamicum. Обзор посвящен анализу основных стратегий разработки продуцентов L-валина на базе C. glutamicum. Рассмотрены различные аспекты биосинтеза L-валина у коринебактерий: биохимия, стехиометрия и регуляция процесса, ферменты и соответствующие им гены, системы экспорта и импорта, связь биосинтеза L-валина с центральным метаболизмом клетки. Выявлены ключевые генетические элементы для создания штаммов-продуцентов на основе C. glutamicum. Описано использование метаболической инженерии для усиления реакций биосинтеза L-валина и уменьшения образования побочных продуктов. Показаны перспективы усовершенствования штаммов с точки зрения повышения их продуктивности и улучшения технологических характеристик. Информация, представленная в обзоре, может быть использована при получении продуцентов других аминокислот с разветвленной боковой цепью – L-лейцина и L-изолейцина, а также D-пантотената.

## Introduction

L-Valine is a proteinogenic branched-chain amino acid (BCAA), which also include L-leucine and L-isoleucine (hereinafter referred to as valine, leucine, isoleucine). These are essential amino acids that are not synthesized in humans or animals and must be present in the diet. Therefore, these amino acids are mainly used in the animal feed industry and as a dietary supplement for humans (Karau, Grayson, 2014). The former is particularly relevant to the global task of intensifying livestock production. Adding valine to feeds, either alone or mixed with other BCAAs, leads to improved meat quality and quantity in pigs and broiler chickens, increased egg production in chickens, increased lactation, milk fat content and appetite in pigs (Zheng et al., 2017; Che et al., 2021; Jian et al., 2021). A balance between different BCAAs, however, must be maintained, as its disruption can reduce the observed beneficial effects (Holen et al., 2022).

In addition to the livestock and food industries, BCAAs find their application in pharmacology and medicine. BCAAs not only serve as building blocks for proteins, but also participate in the regulation of protein and energy metabolism, their consumption increases exercise tolerance and accelerates fatty acid oxidation (Kainulainen et al., 2013). They are useful as supplements for chronic liver disease (Kawaguchi et al., 2011) and for stimulating macrophage phagocytosis of multidrug-resistant bacterial pathogens (Chen et al., 2017). As with feed additives, when using BCAAs for food and drug production their concentration should be chosen carefully. Excess BCAA in human plasma increases the risk of several diseases, including type 2 diabetes, metabolic syndrome, obesity, hypertension, and cardiovascular disease (Holeček, 2018; Dimou et al., 2022), but has little effect on athletes who are characterized by high physical activity (Shou et al., 2019).

Amino acids account for 62.3 % of the global feed supplement market, which is projected to be $34.2 billion in 2022. L-lysine and L-methionine (hereinafter referred to as lysine, methionine) are the most in demand; the valine market is one of the fastest growing, along with L-threonine (hereinafter referred to as threonine) and L-tryptophan. Consumption of feed amino acids is concentrated in Europe, USA and China; Russia’s share is less than 2 %, but shows a growing trend: from 2016 to 2017 the increase was 2.9 % (https://agri-news. ru/zhurnal/2018/32018/ekonomika-menedzhment-ryinki/ ryinok-kormovyix-aminokislot.html). Currently, all valine on the Russian market is imported from China, one of the main producers of this amino acid.

Amino acids can be isolated from natural protein sources, obtained by chemical synthesis, as well as by a microbiological method based on the use of strain-producers. The latter option has important advantages: it allows to use renewable raw material resources and to produce biologically active L-enantiomers of amino acids separately, rather than mixed with D-enantiomers, and is therefore used by leading valine producers (D’Este et al., 2017).

Amino acid producers are developed from Escherichia coli and Corynebacterium glutamicum. E. coli is a thoroughly studied bacterium for which an extensive toolkit of genetic modification is available. Due to that fact producer strains were previously derived mainly from E. coli. However, strains of C. glutamicum created by selection were also used. The history of their use for amino acid production goes back more than 60 years (Leuchtenberger et al., 2005). In recent decades, having made considerable progress both in understanding the metabolism of C. glutamicum and in improving methods for modifying their genome, developers of producer strains have increasingly begun to favor Corynebacteria.

Corynebacteria are nonpathogenic, GC-rich gram-positive bacteria, which, unlike E. coli, do not form endotoxins that cause allergic reactions in higher organisms. They are also characterized by flexible cellular metabolism, genetic stability, stress tolerance, including resistance to high concentrations of carbon sources and metabolites, and the ability to synthesize the target product when growth stops (Baritugo et al., 2018). Valine produced by fermentation using C. glutamicum strains is now recognized as safe (non-toxic and non-carcinogenic) for use as a food and feed additive and for other biological purposes (Kang et al., 2020).

This review presents the main strategies for increasing valine production by C. glutamicum cells. It also summarizes the achievements in the creation of valine-producing strains. In addition to obtaining valine, some aspects of obtaining isoleucine, leucine, and D-pantothenate (hereinafter, pantothenate) are also discussed because the biosynthesis of these compounds involves the same metabolic precursors, cofactors, and enzymes as does valine biosynthesis.

## Valine biosynthesis in C. glutamicum and mechanisms of regulation of this process

**Fig. 1.**
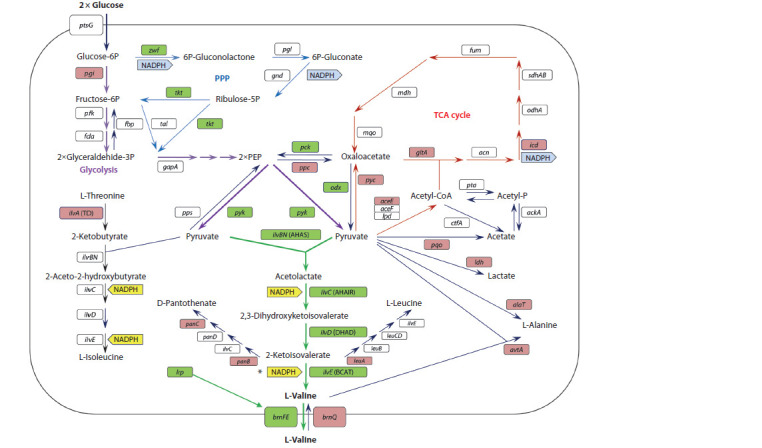
Biosynthesis of valine and related metabolic pathways in C. glutamicum cells. The genes whose increased expression leads to an increase (green) or decrease (red) in valine production are highlighted. A detailed description and transcript of the abbreviations are given in the text. An asterisk marks the reaction in which NADPH is used indirectly (see explanations in the text).

Valine (2-amino-3-methylbutyric acid) is synthesized from pyruvate (pyruvic acid) through four consecutive reactions involving (Fig. 1): 1) condensation of two pyruvate molecules to form acetolactate, catalyzed by acetolactate synthase (AHAS); 2) NADPH-dependent conversion of acetolactate to 2,3-dihydroxyketoisovalerate, catalyzed by acetolactate reductoisomerase (AHAIR); 3) conversion of 2,3-dihydroxyketoisovalerate to 2-ketoisovalerate catalyzed by dihydroxyacid dehydratase (DHAD); 4) NADPH-dependent formation of valine from 2-ketoisovalerate catalyzed by BCAA transaminase (BCAT) and several other transaminases (Yamamoto et al., 2017).

During synthesis, 2 mol of pyruvate and 2 mol of reducing equivalents in the form of reduced nicotinamide dinucleotide phosphate (NADPH) are consumed to produce 1 mol of valine. Pyruvate is formed from phosphoenolpyruvate (PEP) in glycolysis, which converts 1 mol of glucose to 2 mol of pyruvate. The main source of NADPH in Corynebacteria is the pentose-phosphate pathway (PPP) (Marx et al., 1997).

2-Ketoisovalerate is also a precursor of leucine and pantothenate (Park, Lee, 2010). In most microorganisms, including C. glutamicum, the same four enzymes catalyze isoleucine biosynthesis from pyruvate and 2-ketobutyrate. The latter is formed from threonine by threonine dehydratase (TD). Thus, the processes of biosynthesis of all three BCAAs (valine, leucine, and isoleucine) are closely linked. The synthesized BCAAs are removed from the cell by one export system, BrnFE (Lange et al., 2012).

**Fig. 2.**
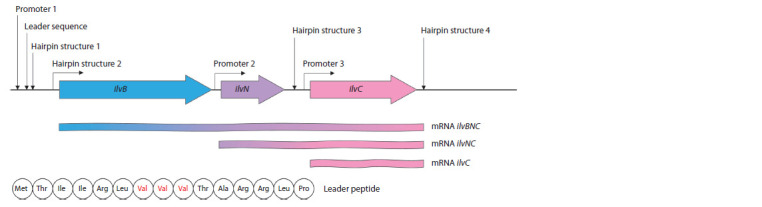
Organization of the ilvBNC operon in C. glutamicum and regulation of its expression (adapted from the review (Wang et al., 2018)).

A schematic of valine biosynthesis and related metabolic pathways in C. glutamicum is shown in Fig. 1. The key enzyme in the biosynthesis pathway of valine and other BCAAs is acetolactate synthase AHAS, which catalyzes the formation of either acetolactate from two pyruvate molecules (in valine and leucine biosynthesis) or 2-aceto-2-hydroxybutyrate from pyruvate and 2-ketobutyrate (in isoleucine biosynthesis). In contrast to E. coli, only one form of the AHAS enzyme was found in C. glutamicum (Keilhauer et al., 1993), a tetramer consisting of two catalytic and two regulatory subunits (Liu et al., 2016). The catalytic and regulatory subunits of AHAS are encoded by the ilvB and ilvN genes, respectively. Together with the ilvC gene encoding the acetolactate reductoisomerase AHAIR, these two genes form the operon ilvBNC with two additional promoters within it. Expression from the three promoters leads to the formation of transcripts of different lengths (Fig. 2). The ilvC gene is transcribed as part of all mRNAs; its expression efficiency is the highest among the three genes (Keilhauer et al., 1993; Morbach et al., 2000).

The expression of the operon ilvBNC is thought to be controlled by the mechanism of transcription attenuation, which is realized through the formation of secondary RNA structures (hairpins) on the transcript, i. e., transcription terminators that arise in the regulatory region in the presence of high concentrations of BCAA (see Fig. 2). The regulatory region is upstream of the ilvB gene; in addition to the sites responsible for hairpin formation, it also encodes a leader peptide (25 amino acids) enriched with isoleucine (2), valine (3), and leucine (2) residues. It is assumed that this peptide is a sensor element of the regulatory system: when the concentration of any of the BCAAs in the cells is low, its translation is slowed down, resulting in no formation of terminator hairpin.

When one or more BCAA were lacking, the expression of operon ilvBNC doubled; replacement of valine residues in the leader peptide with alanine residues led to loss of valine effect on expression (Morbach et al., 2000). A significant increase in the expression of the operon ilvBNC in the presence of 2-ketobutyrate was observed (Eggeling et al., 1987; Keilhauer et al., 1993; Morbach et al., 2000). The mechanism of this regulation has not been investigated.

AHAS activity is strictly inhibited by valine (Ki = 0.9 mM) as well as leucine (Ki = 6.0 mM) and isoleucine (Ki = 3.1 mM) by a feedback mechanism through amino acid attachment to the regulatory subunit of the enzyme (Eggeling et al., 1987; Morbach et al., 2000; Leyval et al., 2003; Elišáková et al., 2005) and is also competitively inhibited by 2-ketoisovalerate (Krause et al., 2010a). Regardless of the number of BCAAs present (one, two, or all three), the degree of inhibition of AHAS activity does not exceed 57 % (Elišáková et al., 2005).

It should be noted that AHAS has lower substrate specificity towards pyruvate (Km = 8.3 mM) (Leyval et al., 2003) than towards 2-ketobutyrate (Km = 4.8 mM) (Eggeling et al., 1987), therefore, all other conditions being equal, the reaction of pyruvate condensation with 2-ketobutyrate leading to isoleucine synthesis is preferred.

As for AHAIR (product of the ilvC gene), which catalyzes the isomerization step and the conversion of acetolactate to 2,3-dihydroxyketoisovalerate and 2-aceto-2-hydroxybutyrate to 2,3-dihydoxy-3-methylvalerate in the isoleucine synthesis pathway, its activity depends on the presence of NADPH and is inhibited by the feedback mechanism of valine and leucine, but not isoleucine (Leyval et al., 2003; Lee et al., 2019).

There is little information about the regulation of the activity of the enzymes controlling the third and fourth, final, steps of valine synthesis in C. glutamicum, as well as about the regulation of the genes encoding their structure. It is only known that the activity of dihydroxyacid dehydratase DHAD (product of the ilvD gene) is weakly inhibited by valine and leucine and not inhibited by isoleucine (Leyval et al., 2003), and the activity of transaminase BCAT (product of the ilvE gene) depends on NADPH availability. The donor amino group in the transamination reaction is L-glutamate (hereafter referred to as glutamate), which is converted to 2-ketoglutarat; NADPH is required for glutamate regeneration by glutamate dehydrogenase. It has been shown that the reaction catalyzed by glutamate dehydrogenase is the main reaction of nitrogen assimilation under conditions of ammonia excess, which usually take place in amino acid production processes (Burkovski, 2003).

It has also been shown that alanine/valintransaminase (a product of the avtA gene) is involved in valine biosynthesis. Alanine/valintransaminase uses L-alanine (hereafter referred to as alanine) or α-aminobutyrate as an amino group donor instead of glutamate (Leyval et al., 2003).

Analysis of the dynamics of changes in the concentrations of the metabolites of valine biosynthesis using a kinetic model in C. glutamicum strain ATCC 13032 ΔpanBC ΔilvA pJC1ilvBNCD showed that the rate-limiting sites in this chain are 1) reactions catalyzed by the AHAS and BCAT enzymes and 2) transport of valine from cells by BrnFE (Magnus et al., 2009).

## Creation of valine-producing strains based on C. glutamicum

The information obtained so far on the biochemical, genetic, and regulatory aspects of valine biosynthesis in C. glutamicum suggests that the barriers to increasing valine production in this microorganism are:

– negative regulation of AHAS activity by valine, leucine, isoleucine, and 2-ketoisovalerate (retroinhibition); – low substrate specificity of AHAS to pyruvate; – negative regulation of ilvBNC operon expression by BCAA; – consumption of pyruvate for synthesis of isoleucine, leucine, and pantothenate; and consumption of 2-ketoisovalerate for synthesis of the latter two compounds; – expenditure of pyruvate and its precursor FEP, key metabolites of glycolytic processes, in cell energy metabolism and carboxylic acid synthesis, as well as in alanine formation; – necessity of NADPH for the second and fourth reactions of valine biosynthesis; – low efficiency of the BCAA BrnFE export system with respect to valine.

In the following, we will review the approaches to overcome these obstacles used in the creation of valine-producing strains based on C. glutamicum (information on the strains is presented in the Table).

**Table 1.**
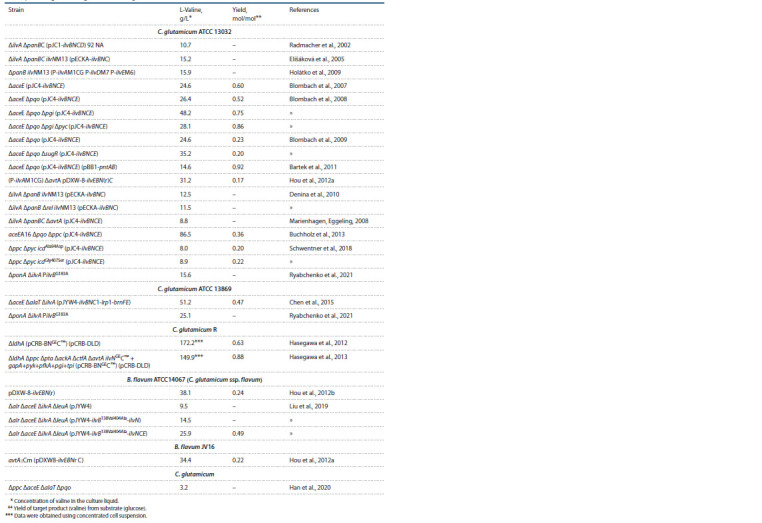
Valine-producing strains engineered from C. glutamicum

## Enhancement of valine biosynthesis reactions

Increase in AHAS activity. There are several approaches to increasing AHAS activity when creating valine-producing strains. The key one is modification of the ilvN gene, which eliminates retroinhibition of the enzyme. A number of mutations in the sequence of the ilvN gene have been found to weaken the effect of BCAA on AHAS activity. These mutations include substitutions of three amino acids, Gly20Asp, Ile21Asp, and Ile22Phe, in the IlvN regulatory subunit (Elišáková et al., 2005). The Ile22Phe substitution showed the best effect in this series, which was later used in other studies (Hou et al., 2012a, b). Similar effects were demonstrated for mutations leading to Ala42Val, Ala89Val, and Lys136Glu substitutions in the small subunit. The double Ala42Val-Ala89Val mutation resulted in almost complete resistance of the enzyme to inhibition by all three BCAAs (Guo et al., 2014).

Enhancement of AHAS substrate specificity with respect to pyruvate. This approach is related to the possibility of modifications of the catalytic subunit IlvB of AHAS that increase the affinity of the enzyme for pyruvate. Reliable data on suitable mutations are scarce. A mutation was found in the ilvB gene that leads to a replacement of alanine for valine at position 138 of the large AHAS subunit. This mutation has made possible a 2.5-fold increase in valine production (Liu et al., 2019). It is assumed that this substitution leads to a change in the substrate specificity of AHAS with respect to pyruvate. The molecular mechanism of action of the mutation remains unclear.

Other mutations in the ilvB gene of the catalytic subunit of AHAS leading to an increase in the enzyme activity toward valine production are also known (Chen et al., 2015; Guo et al., 2015). These other mutations have not yet found practical application.

The modified AHAS enzyme can be introduced into C. glutamicum cells in two ways: either the cells are transformed with a plasmid carrying a mutant gene (Hasegawa et al., 2012; Hou et al., 2012b; Buchholz et al., 2013) or appropriate changes are made in chromosomal DNA (Bartek et al., 2010; Hasegawa et al., 2013). Such manipulations result in a 2–3- fold increase in valine production. The use of autonomous expression plasmids also makes it possible to increase AHAS activity by introducing additional copies of the ilvBN genes or the entire ilvBNC operon into cells. The latter leads to an increase in the activity of not only AHAS, but also AHAIR.

Overcoming the negative effect of BCCA on the expression of the ilvBNC operon. The most rational approach to solve this problem is overexpression of the ilvBNC operon using expression plasmids. At present, overexpression of the ilvBNCDE genes, in various combinations, is performed using constructs with strong constitutive promoters. These include, for example, promoters of superoxide dismutase (Psod ) and elongation factor Tu (Ptuf ) genes and a synthetic construct based on trp and lac operon promoters (Ptac) (Tarutina et al., 2016; Wei et al., 2018; Li et al., 2020b; Wang et al., 2020; Zhang et al., 2021). Other efficient promoters have also been described (Tarutina et al., 2016; Wei et al., 2018; Li et al., 2020b). Modifications of this type lead to an increase in valine production by about 60 % (Wei et al., 2018).

Optimization of the activity of DHAD and BCAT, which catalyze the last steps of valine biosynthesis, is provided by overexpression of the genes encoding these enzymes (ilvD and ilvE, respectively), which is usually achieved by gene amplification on plasmids (see Table). For valine production, it is especially important to increase BCAT activity because this enzyme catalyzes the rate-limiting step of biosynthesis (Magnus et al., 2009).

## Minimizing the formation of byproducts

Enzymes of the valine biosynthesis pathway are involved in the formation of other metabolites such as isoleucine, leucine, and pantothenate (see Fig. 1). Consequently, activation of these enzymes and increased expression of the genes encoding them increase the yield of all the above compounds. This leads to contamination of the target product as well as a decrease in the availability of cofactors, intermediates, and the enzymes themselves for valine production. As a result, it also leads to a lower yield. Minimizing the formation of byproducts when creating strain-producers requires suppression of the relevant metabolic pathways while preserving the strains’ ability to grow on poor media

Minimization of isoleucine formation. As noted above, the synthesis of isoleucine (2-amino-3-methylpentanoic acid) is catalyzed by the same enzymes that are involved in valine biosynthesis and begins with the condensation of pyruvate and 2-ketobutyrate (see Fig. 1). The obvious way to minimize isoleucine formation is to decrease the concentration of 2-ketobutyrate in cells, the interaction of which with pyruvate determines the direction of further reactions. 2-ketobutyrate is formed from threonine by the threonine dehydratase TD, which is encoded by the ilvA gene (Cordes et al., 1992). The threonine dehydratase is negatively allosterically regulated by isoleucine and positively regulated by valine (Möckel et al., 1992).

The most common modification of this gene in the creation of valine-producing strains is its inactivation by deletion (ΔilvA). Most strains were obtained using this modification (see Table). It results in the appearance of the strains’ ability to produce valine or a significant increase in the existing production. In this case, isoleucine auxotrophy occurs, requiring the addition of isoleucine to the cultivation medium, which complicates the production process and may increase the cost of production. In a number of studies to create valine-producing strains, instead of complete inactivation of the ilvA gene, a directed modification of its promoter was performed. This has resulted in a decrease in gene expression, isoleucine bradytrophy, and, as a consequence, increased production of valine (Holátko et al., 2009; Hou et al., 2012a).

Another target for modifications aimed at reducing isoleucine biosynthesis is AHAS. A variant modification of the enzyme’s catalytic subunit that increases its specificity toward pyruvate and redirects cellular resources toward valine production (Liu et al., 2019) is described above.

Minimization of leucine and pantothenate formation. 2-ketoisovalerate is a precursor not only to valine but also to leucine and pantothenate (see Fig. 1). The synthesis of leucine (2-amino-4-methylpentanoic acid) from 2-ketoisovalerate is controlled by the leuA, leuB, and leuCD genes localized in different regions of the chromosome. It is known that leuB and leuCD are subject to the control of the LtbR transcriptional repressor, while leuA regulation seems to involve the mechanism of attenuation of transcription (Wang et al., 2019a). A modification to preserve 2-oxoisovalerate for valine biosynthesis at the expense of decreased leucine biosynthesis was carried out by J. Holátko and colleagues (2009) by reducing the expression of the leuA gene by replacing the native promoter with a weaker one. The result was a 50–70 % increase in valine production, which is comparable to the effect observed when the expression of the ilvA gene is weakened.

The synthesis of pantothenate (amide of β-alanine and pantoic acid) from 2-ketoisovalerate is controlled by the panB and panC genes, which form one operon (Sahm, Eggeling, 1999), and the panD gene which is located separately in the genome (Dusch et al., 1999). It was noted that the carbon flux going to valine biosynthesis is 10 times higher than the flux going to pantothenate biosynthesis, even in the strain with enhanced expression of panBC (Chassagnole et al., 2002). However, inactivation of the panB gene or the entire panBC operon has a favorable effect on valine production, even though it leads to pantothenate auxotrophy in strains. This inactivation allows valine production to appear in wild-type strains and to increase valine production in valine-producing strains by more than 30 % or even 50 % (Radmacher et al., 2002; Holátko et al., 2009).

## Increasing availability of precursors and cofactors

Increasing availability of pyruvate. Pyruvate, the centerpiece of carbon and energy metabolism in all organisms, is a precursor not only to BCAA and pantothenate, but also to many other compounds, including components of the tricarboxylic acid cycle (TCA cycle) as well as lactate and alanine (see Fig. 1). Efficient production of valine requires maintaining a pool of pyruvate in the cells and, therefore, enhancing pyruvate formation reactions and/or reducing its “off-target” consumption. Pyruvate, which synthesizes 2 mol of reduced nicotinamide dinucleotide (NADH), is a product of glycolysis (Wieschalka et al., 2012). However, glycolytic enzyme activity is generally not increased in the development of valine producers, except for the microaerobic process (see below). The main approach is to reduce the outflow of pyruvate, and its precursor PEP, into other pathways.

One of the main pathways of pyruvate outflow is the TCA cycle. This process becomes less active in the late stages of growth, which could be used to create favorable conditions for valine production. Indeed, a decrease in the growth rate of C. glutamicum is accompanied by an increase in pyruvate levels in cells and an increase in valine (Ruklisha et al., 2007). In valine-producing strains that are auxotrophic for isoleucine and pantothenate, growth of cultures can be controlled by changing the amount of supplementation with these substances. Growth restriction also leads to increased productivity (Bartek et al., 2008).

Involvement of pyruvate and PEP in the TCA cycle occurs both through conversion of both compounds to oxaloacetate (OA) and of pyruvate to acetyl-CoA directly or through acetate and acetyl-phosphate (see Fig. 1). As a rule, increasing the production of valine as well as pyruvate itself is attempted through reducing the activity of the pyruvate dehydrogenase complex (PDHC), which catalyzes the oxidative decarboxylation of pyruvate to acetyl-CoA. In C. glutamicum, this complex consists of three subunits, E1, E2, and E3, encoded by the aceE, aceF, and lpd genes, respectively (Eikmanns, Blombach, 2014). Inactivation of the aceE gene by deletion (ΔaceE) is one of the most frequent steps in creating a valine producer (see Table). The resulting strains require the addition of acetate in minimal medium, but the level of valine production increases manifold. Metabolomic analysis showed that inactivation of aceE in wild-type C. glutamicum leads to a 13-fold increase in the pyruvate pool in cells (Blombach et al., 2007).

A characteristic feature of C. glutamicum strains devoid of PDHC is the production of valine in the absence of cell growth. Increased glucose utilization rate was achieved by adding maltose instead of glucose, using ethanol instead of acetate, or inactivating the transcriptional regulator SugR (Blombach et al., 2009; Krause et al., 2010b). SugR in C. glutamicum is responsible for acetate-mediated repression of the ptsG, ptsI, and ptsH genes encoding the enzymes of the phosphotransferase system (PTS). PTS ensures the conjugated processes of sugars transport into the cell and their phosphorylation (Engels, Wendisch, 2007). However, because of PDHC deficiency, all strains still needed acetate or ethanol, which is then also converted to acetate as an additional carbon source.

To overcome this need, the native aceE gene promoter was replaced with mutant variants from a previously established promoter library based on the dapA gene promoter (Vasicová et al., 1999). This allowed to obtain a series of C. glutamicum strains with gradually decreased PDHC activity as well as gradually decreased growth rate on medium containing glucose as the only carbon source. Transformed with the pJC4-ilvBNCE plasmid, these strains produced valine and did not require acetate as an additional carbon source (Buchholz et al., 2013). A growth-dependent promoter of the aldehyde dehydrogenase gene from C. glutamicum CP (PCP_2836) has been used for the same purposes. This has led to a threefold decrease in aceE transcription levels compared to the native promoter, as well as has had positive effects on both cell growth and valine production (Ma et al., 2018b).

It is also possible to reduce pyruvate consumption in the TCA cycle by decreasing the activity of the cycle itself. For example, suppression of the gene of the transcription factor RamA responsible for the TCA cycle activation has been shown to contribute to efficient pyruvate production (Kataoka et al., 2019).

The conversion of pyruvate to acetate is catalyzed by pyruvate:quinoxidoreductase (product of the pqo gene), the inactivation of which (Δpqo) leads to increased valine production (see Table), but also to impaired growth characteristics of strains. The combination of this modification with inactivation of PEP carboxylase (product of the ppc gene), which catalyzes formation of OA from PEP, resulted in a slight increase in valine production, however, the yield increased by 14 % (Buchholz et al., 2013). It was noted that the valine-producing strain with inactivated aceE and pqo genes grew better and produced more valine on maltose-enriched medium (Krause et al., 2010b).

Another pathway for the outflow of pyruvate is the formation of OA from it under the action of pyruvate carboxylase (product of the pyc gene). Inactivation of pyc in the creation of a valine-producing strain leads to an increase in yield to 0.86 mol of valine per 1 mol of glucose (Blombach et al., 2008). When developing a leucine-producing strain, it was found that, in order to minimize pyruvate outflow, inactivation of pyruvate carboxylase is more beneficial than inactivation of PEP carboxylase (Wang et al., 2020).

Two other pathways of pyruvate consumption in C. glutamicum cells are the processes of lactate and alanine biosynthesis (see Fig. 1). Lactate formation catalyzed by lactate dehydrogenase (a product of the ldhA gene) becomes important in terms of valine production under oxygen deficiency conditions (Hasegawa et al., 2012) and will be discussed further.

Minimization of alanine synthesis is required under all conditions because this process leads not only to untargeted consumption of pyruvate but also to loss of NADPH in the amino group transfer reaction and to unwanted impurities in the final product.

Alanine formation in Corynebacteria is catalyzed by the transaminases AlaT and AvtA, which use glutamate and valine as amino group donors, respectively (Marienhagen et al., 2005; Marienhagen, Eggeling, 2008). It was noted above that AvtA is one of the transaminases involved in valine biosynthesis, but its role, compared with BCAT, is minor

The question of the participation of these transaminases in alanine biosynthesis in C. glutamicum remains open due to the inconsistency of existing data. On the one hand, inactivation of alaT and avtA in the valine-producing strain has been shown to reduce alanine formation by about 80 and 20 %, respectively (Marienhagen, Eggeling, 2008). A significant decrease in alanine synthesis (to less than 0.2 g/L) is observed as a result of the inactivation of both genes (Hou et al., 2012a). These data suggest that the AlaT aminotransferase is the major one, but both enzymes are involved in alanine synthesis. On the other hand, in the proline producer, inactivation of alaT has no effect on alanine levels, whereas inactivation of avtA reduces this level by 48 % (Zhang et al., 2020). Moreover, analysis of the transcriptome of the industrial valine producer line VWB-1 showed that its low level of L-alanine synthesis is not associated with the alaT gene, the transcriptional level of which in this strain is 5.1-fold higher than that in the wild-type strain. It is assumed that a lower level of L-alanine synthesis is due to the lower expression of the gene alr encoding alanine racemase that converts L-alanine to D-alanine (Zhang H. et al., 2018). Thus, it is also impossible to give an unequivocal answer to the question of whether inactivation of one or the other of these two transaminases is more advantageous in terms of valine production.

Increasing availability of NADPH. In C. glutamicum, the main supplier of NADPH is PPP, in which the reduction of NADP+ to NADPH is provided by glucose-6-phosphate dehydrogenase (a heteromultimeric complex wherein one of the subunits is encoded by the zwf gene) and 6-phosphogluconate dehydrogenase (a product of the gnd gene). The activity of both enzymes is negatively regulated by ATP, NADPH, and other metabolites (Moritz et al., 2000). NADPH-dependent decarboxylating malate dehydrogenase (malic enzyme) and isocitrate dehydrogenase play a minor role in the synthesis of NADPH from NADP+ (Bartek et al., 2010; Siedler et al., 2013). The source of NADP+ and, hence, the source of NADPH in C. glutamicum can also be NAD+, which is phosphorylated by NAD kinase (product of the ppnK gene) to form NADP+. This enzyme has been characterized as a polyphosphate-ATP-dependent NAD kinase that uses ATP to phosphorylate NAD+ (Shi et al., 2013).

Theoretical analysis showed that the level of substrate conversion to valine (the yield) significantly depends on the reactions used for NADPH regeneration. The maximum yield, equal to 1 mol of valine per 1 mol of glucose, is obtained without the expenditure of carbon for growth and synthesis of NADPH. If NADPH is provided by isocitrate dehydrogenase activity, the yield is 0.5 mol of valine per 1 mol of glucose. Directing the entire carbon flux into the NADPH-generating PPP results in a much higher yield of 0.86. In this analysis, the main target for the redirection of carbon flux from the TCA cycle to valine biosynthesis appeared to be PDHC. A scenario in which carbon is not consumed for NADPH synthesis at all can be realized by the combined activity of pyruvate carboxylase (or PEP carboxylase), malate dehydrogenase, and malic enzyme, theoretically capable of transferring hydrogen from NADН to NADP+ (Bartek et al., 2010). Such a path- way, designated a transhydrogenase-like shunt, is involved in NADPH formation for anaerobic isobutanol production in C. glutamicum (Blombach, Eikmanns, 2011). Thus, enhancement of PPP and NAD kinase activity are the most obvious ways to increase the NADPH pool in the cell.

From the point of view of the efficiency of the valine biosynthesis process, it is advantageous to combine the enhancement of PPP with some weakening of glycolysis. Indeed, inactivation of the glucose-6-phosphatisomerase gene pgi (this inactivation directs carbon flux from glycolysis to PFP) resulted in more efficient valine production in the C. glutamicum strain ΔaceE Δpqo Δpgi (pilvBNCE), producing 48.0 g/L with a yield of 0.75 mol of valine per 1 mol of glucose (Blombach et al., 2008). Further analysis of this strain showed that inactivation of pgi results in increased intracellular NADPH concentrations and decreased byproduct formation (Bartek et al., 2010). Monitoring cellular NADPH content using NADPH-dependent fluorescence also showed that the C. glutamicum strain carrying Δpgi does accumulate NADPH (Goldbeck et al., 2018).

The growth deterioration observed in Δpgi-strains on medium with glucose has been attributed to a decrease in PTS activity and suggested to be overcome by overexpression of the gene ptsG, which encodes a glucose-specific component of this system (Lindner et al., 2013). For pgi-mutants, enhancement of the alternative glucose transport system by inositol permeases IolT1, IolT2, and the glucokinase PpgK, which was used to produce lysine producer, is also effective (Xu J.Z. et al., 2019).

Another approach to increase the NADPH pool is related to the possibility of changing the specificity of glycolytic enzymes from NAD+ to NADP+. It has been implemented to improve lysine production. Point mutations in the glyceraldehyde- 3-phosphate dehydrogenase gapA gene that changed enzyme specificity resulted in a 35–60 % increase in lysine production (Bommareddy et al., 2014; Xu et al., 2014).

It was noted above that enzymes that synthesize NADPH are susceptible to negative regulation by various metabolites. Therefore, one approach to PPP activation is to introduce into the corresponding genes mutations that increase enzyme activity. Such an approach has been implemented for the zwf and gnd genes in works on methionine, proline, and riboflavin producers. It has indeed led to an increase in the NADPH pool and production levels in cells (Wang et al., 2011; Li et al., 2016; Zhang et al., 2020).

As for NAD-kinase, the studies published to date on enhancing its activity target isoleucine production. These studies indicate that modifications that increase the enzyme activity (point mutations in the ppnK gene, overexpression of the ppnK gene) lead to increased intracellular concentration of NADP+ and NADPH and contribute to enhanced biosynthesis of the target product (Yin et al., 2014; Zhang et al., 2020).

Another attractive possibility for increasing NADPH availability for valine biosynthesis is heterologous expression of transhydrogenase genes, such as pntAB from E. coli, that catalyze NADP+ reduction involving NADH. This possibility was previously used to improve lysine production with C. glutamicum (Kabus et al., 2007). A significant increase in intracellular NADPH concentration was observed when pntAB expression was combined with overexpression of the ppnK gene (Zhan et al., 2019). Introduction of PntAB from E. coli into the valine-producing strain C. glutamicum ΔaceE Δpqo (pJC4ilvBNCE) resulted in a significant decrease in carbon flux in PPP and, consequently, an increase in yield to 0.92. This is the highest yield (Bartek et al., 2011), which is only 8 % below the theoretical maximum of 1 mol of valine per 1 mol of glucose (Bartek et al., 2010).

## Engineering the microaerobic process of valine production

Under oxygen deprivation, C. glutamicum cultures show very poor growth capacity but metabolize sugars to organic acids (Michel et al., 2015; Lange et al., 2018). When byproduct synthesis is suppressed, producer strains adapted to such conditions show higher productivity than strains requiring aeration (Okino et al., 2008; Jojima et al., 2010, 2015; Yamamoto et al., 2013). Valine biosynthesis under normal conditions is an aerobic process because it is carried out by growing cultures actively generating NADPH. For efficient production of valine under oxygen deprivation, strains require a complex modification involving both valine biosynthesis enzymes and glycolysis enzymes. Such a modification was performed by S. Hasegawa and colleagues (2012, 2013).

The C. glutamicum R strain with inactivated lactate dehydrogenase (ΔldhA) and overexpression of the ilvBNCE genes encoding the enzymes of valine biosynthesis was used as the basis for creating strains producing valine under microaerobic conditions. This strain is incapable of producing valine under oxygen deficiency because it has an imbalance of cofactors: 2 mol of NADPH are consumed while 2 mol of NADH are synthesized to produce 1 mole of valine.

The appearance of valine production was achieved by using two approaches. The first approach was to change the specificity of AHAIR from NADPH to NADH by site-directed mutagenesis of the ilvC gene (constructing the ilvC™ gene). The second approach was to replace the NADPH-dependent transaminase BCAT with NAD-dependent leucine dehydrogenase (LeuDH) from Lysinibacillus sphaericus (Hasegawa et al., 2012). The additional introduction of the ilvN gene encoding a mutant AHAS regulatory subunit (ilvN GE) resistant to BCAA inhibition has allowed to produce a C. glutamicum strain (pCRB-BN GEC™)(pDLD)/ΔLDH) that produced 172.2 g/L of valine for 24 h under microaerobic conditions with periodic fermentation, which was more than 20-fold higher than baseline. The yield was 0.63 mol of valine per mol of glucose (Hasegawa et al., 2012).

However, in addition to valine, the cells of this strain accumulated significant amounts of alanine, acetate, and succinate as byproducts. To eliminate their formation and increase the valine yield, additional modifications were introduced into the strain (Hasegawa et al., 2013). Succinate formation via PEP and OA was suppressed by inactivation of the ppc gene, but this resulted in reduced valine synthesis and glucose uptake, as the intracellular NADH/NAD+ ratio increased markedly. To restore the ratio to a level favorable for valine production, three genes involved in acetate synthesis (pta, ackA, ctfA) were inactivated and the expression of five genes (gapA, pyk, pfkA, pgi, tpi) encoding glycolysis enzymes was increased. As a result, valine production increased 9-fold and glucose uptake, 7.6- fold. Since valine biosynthesis became an NADHdependent process, increasing the activity of glycolytic enzymes turned out to be beneficial in terms of accumulating both pyruvate and reducing equivalents.

Decrease in alanine formation was achieved by inactivation of the avtA gene. In addition, the ilvN GE and ilvC™ genes, which were previously expressed on the plasmid, were placed in the chromosome. The valine productivity of the new strain was 149.9 g/L in 24 h of cultivation. The yield reached 0.88 mol of valine per mol of glucose, which was significantly higher than that obtained in the first step (Hasegawa et al., 2013).

It should be noted that in both works, valine synthesis under microaerobic conditions was studied using non-growing cells preconcentrated by centrifugation by a factor of 2 to 3. In this case, the measured valine concentration reached very high values, but the productivity per cell was comparable with that demonstrated in other studies

Replacement of enzyme specificity from NADPH to NADH to adapt the amino acid production process to microaerobic conditions has also been done in the development of E. colibased valine producer (Savrasova, Stoynova, 2019) and C. glutamicum- based leucine and L-ornithine producers (Jiang et al., 2013; Wang et al., 2019b). In all cases, this resulted in an increased yield of the target product.

## The engineering of valine transport

Microorganisms have multiple transport systems that ensure the uptake of desired environmental components by cells and release of metabolites, the excess of which can be toxic(Pérez-García, Wendisch, 2018). The activity of such systems depends on the concentration of the transported substances, so it has long been thought that producing strains’ own regulatory mechanisms are sufficient for excreting the target products effectively (Jones et al., 2015). Transport engineering is complicated by the complexity of its quantification and the fact that specific transporters are not known for each biotechnologically relevant substance. In recent years, however, there have been a growing number of studies showing the effect of directional changes in export and import of the target product on strain productivity (Eggeling, 2016). Valine transporters in Corynebacteria have been detected and characterized, and thus are promising targets for modifications in the creation of producing strains. 

Valine import. The uptake of valine, leucine, and isoleucine in Corynebacteria occurs through a secondary Na+-dependent symport carried out by the only known importer, BrnQ (Ebbighausen et al., 1989). BrnQ exhibits the highest affinity for isoleucine. For valine and leucine, the affinity is 1.7 times lower (Ebbighausen et al., 1989; Tauch et al., 1998). Data on the regulation of BrnQ and the corresponding gene in corynebacteria are extremely scarce. It is known that BrnQ is activated when the intracellular concentration of BCAA is increased (Boles et al., 1993) and that inactivation of the brnQ gene increases isoleucine export from C. glutamicum cells and its production (Xie et al., 2012). It has been noted that a similar modification favors growth and productivity of the isoleucine-producing strain WM001 in the early stages of fermentation (Zhang et al., 2020). The importance of the importer for valine production is confirmed by transcriptome analysis of the industrial producer VWB-1, which showed that the transcription level of the brnQ gene in this strain is lower than that of the wild-type strain (Zhang H. et al., 2018).

**Fig. 3.**
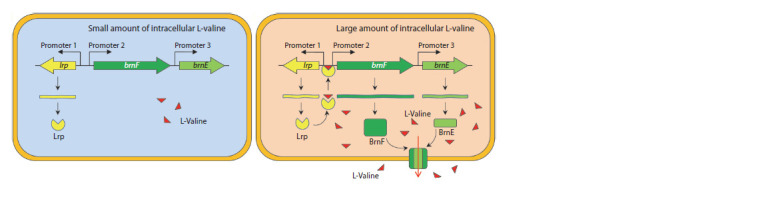
Organization of the brnFE operon in C. glutamicum and regulation of its expression (from the review (Wang et al., 2018)).

Valine export. The BrnFE transport system is responsible for BCAA export from C. glutamicum cells (Eggeling, Sahm, 2003). Amino acids are exported through a secondary H+-dependent process controlled by membrane potential (Hermann, Kramer, 1996). The brnFE transport system is the only known exporter of valine, leucine, and isoleucine in C. glutamicum. It also transports methionine and homoserine, a precursor of methionine, isoleucine, and threonine (Kennerknecht et al., 2002; Trotschel et al., 2005; Yin et al., 2013; Qin et al., 2015; Li et al., 2020a). The brnF and brnE genes encoding, respectively, the large and small subunits of the transport system, are organized into a single operon controlled by the transcriptional regulator Lrp (leucine responsive protein) (Kennerknecht et al., 2002; Lange et al., 2012). Homologues of Lrp, first discovered and characterized in E. coli, are present in the genomes of various prokaryotes and regulate genes involved in amino acid metabolism (Brinkman et al., 2003). In C. glutamicum, the lrp gene is located divergently upstream of the brnFE operon. By binding to BCAA or methionine, Lrp becomes active and, in turn, activates the brnFE promoter (Kennerknecht et al., 2002; Lange et al., 2012) (Fig. 3). The effect of cellular amino acid concentration on Lrp activity decreases in the series leucine > methionine > isoleucine > valine (Lange et al., 2012).

A study of industrial leucine and valine producers confirms that a high level of amino acid production either correlates with a high level of operon brnFE expression (Vogt et al., 2014; Zhang H. et al., 2018) or is associated with an increased lrp and brnFE gene copy number (Ma et al., 2018a).

Analysis of the effect of operon brnFE on valine production showed that its overexpression does not affect the growth of C. glutamicum cells and increases valine production by about 2–3 times (Chen et al., 2015). Overexpression of brnFE has a similar effect on the production of isoleucine, methionine, and homoserine (Qin et al., 2015; Li et al., 2020a; Zhang et al., 2021). The maximum effect on isoleucine production was obtained when lrp and brnFE expression were simultaneously enhanced (Yin et al., 2013).

However, it was found that, unlike brnFE, overexpression of the lrp gene suppresses cell growth (Chen et al., 2015), although it also significantly increases valine production. The negative effect was counterbalanced by the use of a weakened mutant form of this lrp1 gene found in the VWB-1 strain. Overexpression of lrp1 in the wild-type C. glutamicum strain resulted in a 16-fold increase in valine production, from 1.9 to 30.2 mmol/L per 96 h of cultivation. The combination of lrp1 and brnFE overexpression enhanced the effect. Isoleucine production was not significantly affected by such manipulations, from which the authors concluded that isoleucine is a less suitable substrate for brnFE than valine (Chen et al., 2015). Simultaneous amplification of the expression of both genes, lrp and brnFE, combined with overexpression of the ilvBNC genes and inactivation of aceE, alaT, and ilvA, resulted in a strain that produced 437 mM (51 g/L) valine when fermented with feeding (Chen et al., 2015).

Thus, modifications of BCAA transport systems aimed at reducing the influx of amino acids into the cell and increasing their secretion from the cell have a positive effect on the production of the amino acids (Xie et al., 2012).

## Conclusion

In recent years, interest in the use of valine as a feed additive has increased significantly. In the Russian Federation alone, the consumption of valine has increased almost 10-fold over the past five years, reaching 5,000 tons per year. Modern industrial production of valine is based on microbiological synthesis using renewable plant raw materials and producing strains with a modified genetic program. The efficiency of amino acid production largely depends on the productivity of the producer strains, which are a key element of the entire process chain. Although significant progress has been made in the creation of producing strains (see Table), the creation of new strains with unique characteristics is still relevant.

It is worth noting that the recently developed processes with reduced aeration have a higher potential compared to the traditional aerobic processes of valine production. However, it should be noted that such processes are biphasic: in the first phase, biomass is produced aerobically, while in the second phase, valine biosynthesis occurs under microaerobic conditions. Currently, the two-phase processes show low efficiency, and more research in this area is required.

Nowadays, the main approach to creating valine-producing strains, which has replaced random mutagenesis, is rational metabolic engineering aimed at enhancing the valine biosynthesis process and minimizing the formation of byproducts. In recent years, this approach has been actively enriched by the application of systems engineering and synthetic biology methods. The combined analysis of “omics” data expands our knowledge of the metabolic and regulatory processes of C. glutamicum and allows us to develop new strategies for creating producers of valine and other amino acids. The recent emergence of rapid genome editing systems that speed up the process of obtaining new strains should help to implement these strategies.

Further progress in the creation of producer strains will involve a shift from studying the properties of a cell population to studying the properties of individual cells (Harst et al., 2017; Hemmerich et al., 2018; Pérez-García et al., 2018), as well as extensive application of computer modeling (Koduru et al., 2018) and using new knowledge about gene expression regulation (Dostálová et al., 2017; Shi et al., 2018; Zhang S. et al., 2018; Xu N. et al., 2019).

The approaches perfected in the creation and improvement of valine producers can be used to create producers of other BCAA and pantothenate, the substances that also have a significant market potential.

## Conflict of interest

The authors declare no conflict of interest.
